# Association between Oral Frailty and Physical Frailty among Rural Middle-Old Community-Dwelling People with Cognitive Decline in Taiwan: A Cross-Sectional Study

**DOI:** 10.3390/ijerph19052884

**Published:** 2022-03-01

**Authors:** Ya-Wen Kuo, Jiann-Der Lee

**Affiliations:** 1 Department of Nursing, College of Nursing, Chang Gung University of Science and Technology, Puzi 613, Taiwan; 2 Department of Neurology, Chang Gung Memorial Hospital Chiayi Branch, Puzi 613, Taiwan; jdlee540908@gmail.com; 3 Department of Medicine, Chang Gung University, Taoyuan 333, Taiwan

**Keywords:** oral frailty, physical frailty, rural middle-old people, cognitive decline

## Abstract

This cross-sectional study was conducted to clarify the association between oral frailty (OF) and physical frailty (PF). In total, 308 Taiwanese middle-old (75–84 years) community-dwelling people with cognitive decline were recruited from random rural community health care centers. Eight items were considered for the evaluation of their OF status. The Study of Osteoporotic Fracture index was used for frailty assessment, which revealed that 22% of the participants had PF. In an adjusted model, PF was significantly associated with the OF subdomains of difficult to eat hard food (*p* = 0.009), choking (*p* = 0.008), denture use (*p* = 0.008), and inability to chew hard food (*p* = 0.001); and high risk of OF (OR = 3.03; *p* = 0.002). After eight steps with elimination of the least significant independent variable, age (*p*= 0.008), self-reported health status of fair (*p* = 0.000) and poor (*p* = 0.000), mild cognitive impairment (*p* < 0.001) and denture use (*p* = 0.011) were found to be the indispensable predictors for PF. The logistic regression model with 5 indispensable variables had a predictive value of 78.2%. Longitudinal analyses are needed to examine whether OF is a risk factor for PF onset.

## 1. Introduction

Population aging is a global phenomenon, and the population with coexisting frailty and cognitive decline is growing worldwide [1]. Most people in this population are aged ≥75 years [2]. Toward the end of 2020, mild cognitive impairment and dementia were reported in 684,108 (18.06%) and 291,961 (7.71%) people, respectively, aged ≥65 years in Taiwan [3]. The higher the age, the higher the prevalence rate, and with every 5 years’ increase in age, a tendency of the prevalence rate to double was noted; the prevalence rate of dementia in the middle-old population (75–84 years) was 20.12% in 2020 [4]. In a community-based prospective cohort study in Taiwan, the prevalence of mild cognitive impairment in the rural community was much higher than that in the urban community [5].

Frailty is defined as physical decline associated with aging, which is characterized by symptoms that have significant adverse health outcomes [6]. Fried defined frailty as an aging-related syndrome in which three or more of the following criteria are present: unintentional weight loss, self-reported exhaustion, weakness (grip strength), slow walking speed, and low physical activity [7]. A systematic review and meta-analysis revealed that physical frailty (PF) is highly prevalent in older patients with cognitive decline in the community, with a pooled prevalence of 31.9% [8]. Frailty assessment in the elderly population in the community setting is challenging. The Study of Osteoporotic Fracture (SOF) index is used to assess the frailty status of older people in the community, which is based on the presence of at least two of the following components: weight loss, exhaustion, and low mobility [9]. A systematic review reported that impairments in swallowing and masticatory functions in older adults are associated with weight loss and malnutrition [10]. Malnutrition is associated with cognitive impairment or functional loss, and people with an inadequate nutritional status are predisposed to cognitive frailty and PF [11]. PF and cognitive impairment may coexist among patients with dementia, thus increasing the risks of disability, adverse health outcomes, and death [12].

Oral frailty (OF) is a decline in oral function, oral structure, and oral health [13]. A study in Japan reported that poor oral status could be determined based on chewing ability, the number of natural teeth, tongue pressure, articulatory oral motor skill, and subjective difficulties in eating and swallowing, and OF was defined as coexisting poor status in at least three of these six measures [14]. A systematic review reported that oral health status based on four factors, namely deterioration of oral motor skills, chewing and swallowing, saliva disorders, and oral pain, was associated with age-related gradual loss of oral function and a decline in cognitive function and physical function [15].

Approximately 16% of older adults had OF at baseline, which was significantly associated with 2.4-, 2.3-, and 2.2-fold increased risks of PF, disability, and mortality, respectively [14], but the relationship among OF, OF subdomains, and the SOF index of PF of middle-old people with cognitive decline in rural communities is unexplored. This study investigated the OF and OF subdomains in a rural population with cognitive decline with different frailty levels to assess their association with participant characteristics. A straightforward, rapid, and frequent person-centered care assessment is crucial for middle-old elderly people with cognitive decline in the rural community; thus, the PF assessment tool with SOF was used in this study. We explored the relationship between OF and SOF frailty criteria to confirm empirical evidence for the early detection of OF and PF in middle-old people with cognitive decline living in rural communities.

## 2. Materials and Methods

### 2.1. Study Population

A cross-sectional study was conducted at 18 randomly selected community-based cognitive decline care centers from 29 communities of 18 townships in Chiayi County, Taiwan. We choose a community-based care center per township. In 2021, >8% of the population with cognitive decline in Chiayi County was aged ≥75 years. First of all, we contacted the person in charge of each community care center to agree on the time of the briefing session. Then, we recruited elderly people as participants in this study from September 2019 to June 2020 after obtaining approval from the authorities in charge of the community-based cognitive decline care centers and the caregivers of potential participants. A sample size of 365 participants was included. The inclusion criteria were (1) age 75–84 years and (2) ability to communicate in Taiwanese or Mandarin. The exclusion criterion was the inability to cooperate because of mental disorders. This study used a self-administered structured questionnaire, interviews, and direct assessment with caregivers of participants for data collection. The participants’ sociodemographic and health characteristics were examined, including age, gender, education level, number of natural teeth, chronic disease history, self-reported health status (good, fair, or poor), and cognitive function. Furthermore, the medical history of chronic disease was determined by assessing hypertension, diabetes mellitus, heart disease, and Parkinson’s disease. This study followed the principles of the Declaration of Helsinki and was approved by the National Cheng Kung University Research Ethics Committee (Approval No. 108-033). All participants verbally agreed to participate in this study and were included after obtaining informed consent. The anonymity of participants was maintained, and research data were collected using serial numbers instead of participant names in this study.

### 2.2. Clinical Dementia Rating

The clinical dementia rating (CDR) scale is widely used to assess cognitive decline with dementia. The CDR scale contains six domains: memory, orientation, judgment and problem solving, community activities, home and hobbies, and personal care. The scores of the six domains are summed to create a 0–18 “sum of the boxes”. It is rated on a 5-point scale from 0 to 3: no impairment (CDR = 0), questionable dementia (CDR = 0.5), mild cognitive impairment (CDR = 1), moderate cognitive impairment (CDR = 2), and severe cognitive impairment (CDR = 3) [16]. The reliability and validity of the Chinese version of the CDR scale for mild cognitive impairment and dementia among elderly people were evaluated, which showed good internal consistency (kappa = 0.63) [17].

### 2.3. Oral Frailty

The OF checklist proposed by the Japanese Dental Association [18] was the basis for OF assessment. The checklist consists of eight items: (1) find it more difficult to eat hard food now than half a year ago; (2) sometimes choke on tea or soup (choking); (3) use dentures; (4) have concerns regarding oral dryness; (5) go out less frequently now than half a year ago; (6) capable of chewing hard food, such as pickled radish or shredded and dried squid; (7) brush teeth at least twice a day; and (8) visit a dentist at least once a year.

If subjects answered yes to item 1, 2 or 3, two points were given, and if yes to item 4, 5 or 6, one point was given for each answer. When the subjects answered no to item 7, 8 or 9, one point was given for each answer. The maximum score was 11. The OF criteria were defined based on the sum of the scores: 0–2 points indicated low risk, 3 points indicated moderate risk, and >4 points indicated high risk. The validated questionnaire is a useful tool for screening OF [19].

### 2.4. Physical Frailty

In this study, the SOF index was used to assess the PF of elderly people with dementia. SOF is a simple, effective, and rapid three-item frailty screening tool for PF assessment in elderly people [6]. The three items of the SOF index are (1) weight loss of ≥5% during the preceding year; (2) inability to rise from a chair five times without using the arms; and (3) energy loss. Participants with none of these three impairments were considered robust, participants meeting one criterion were considered to have pre-frailty status, and participants meeting two of three criteria were considered to suffer frailty [20].

### 2.5. Statistical Analysis

Statistical analyses were performed using SPSS version 20 (IBM Corp, Armonk, NY, USA). Descriptive statistics were used to stratify participants into three groups based on their frailty level (namely, robust, pre-frailty, and frailty). Continuous variables were presented as mean ± standard deviation, and categorical variables were presented as numbers and percentages. One-way analysis of variance (ANOVA), chi-square, and logistic regression analysis was used. The Kruskal–Wallis test was used when the assumptions of normality for ANOVA were not met. Levene’s test was used for homogeneity of variances. The Welch test was used with unequal sample sizes and when the homogeneity of variance assumptions for one-way ANOVA were violated, the Games–Howell test was used for post hoc analysis. Logistic regression models were used to test the association between OF and PF and to generate odds ratios (ORs) adjusted for the variables of study participants. The occurrence of the event was coded as frailty (including pre-frailty and frailty) and non-frailty. Finally, logistic backward regression analysis was performed, resulting in a final model containing only statistically significant predictors of acceptance. The explanatory independence variables with the full model contained age, number of natural teeth, number of chronic diseases, Parkinson’s disease, self-reported health status, CDR scale, difficulty eating hard food, choking, denture use, xerostomia, inability to chew hard food, visiting the dentist irregularly, and risk of OF. The *p*-value of <0.05 was considered statistically significant.

## 3. Results

In total, 365 middle-old people with cognitive decline were included in this study. After excluding invalid questionnaires, that is, questionnaires with incomplete data, 308 participants were included in this study, for a recovery rate of 84.4%. In total, 231 (75%) participants were women, and 82.1% had an education level of elementary school or below. In total, 215 (69.7%) participants were in pre-frailty (*n* = 147, 47.7%) or frailty (*n* = 68, 22%) stages. Fair or poor health status was expressed by 74.8% of the middle-old people with cognitive decline who were in pre-frailty and frailty stages. Age (*p* = 0.001), natural teeth (*p* < 0.001), Parkinson’s disease (*p* = 0.018), self-reported health status (*p* < 0.001), and CDR scores (*p* = 0.002) at different frailty levels presented significant differences. Compared with the non-frailty population, the frailty population had a higher mean number of chronic diseases. However, there were no significant differences in relation to the number of chronic diseases among the three groups (robust, pre-frailty and frailty). The middle-old people with cognitive decline along with hypertension, heart disease, and Parkinson’s disease comprised >70% of the population with pre-frailty and frailty status (Table 1).

The middle-old people with various frailty levels were compared in terms of their OF. The mean OF score was 4.87 for all populations. Comparing the frailty levels of elderly people, the frailty population had a higher OF mean score than did the robust and pre-frailty populations. The results revealed significant differences in OF abnormal items (*p* = 0.000), OF mean score (*p* = 0.000), difficulty eating hard food (*p* = 0.000), choking (*p* = 0.000), denture use (*p* = 0.000), inability to chew hard food (*p* = 0.000), and visiting the dentist irregularly (*p* = 0.017) for cognitive decline in elderly people with different frailty levels. The risk of OF was significantly different (*p* = 0.000) across different frailty levels, and 80.6% of the elderly people with a high risk of OF were in the pre-frailty and frailty stages (Table 2).

**Figure 1:**
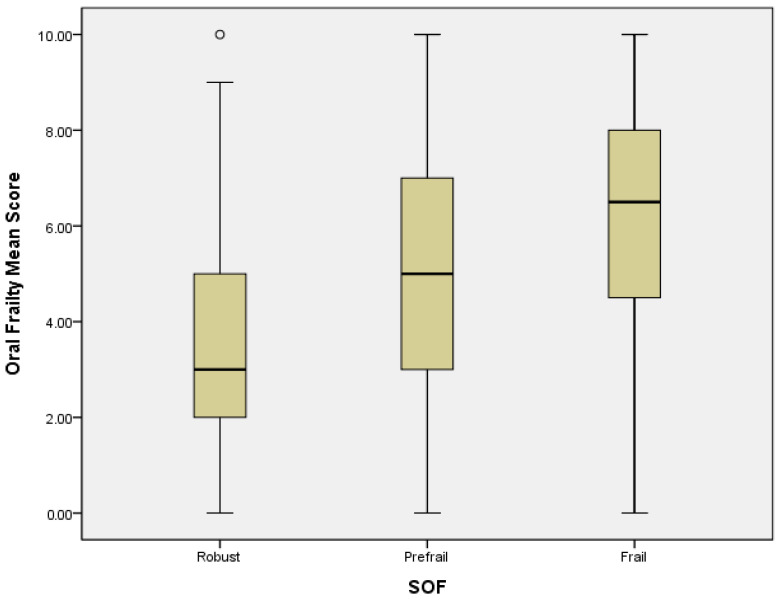
The correlation between OF mean score and PF. Mild outliers are marked with a circle “o”.

**Figure 2:**
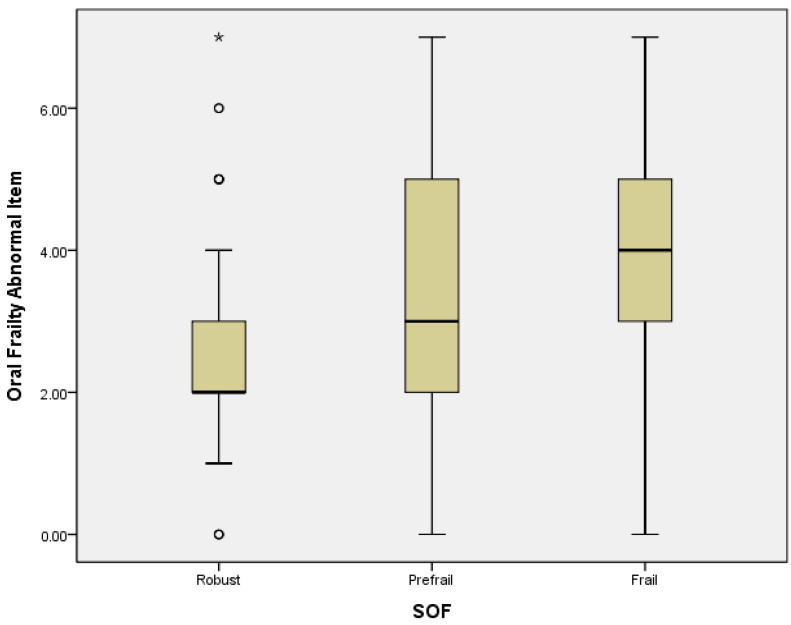
The correlation between mean abnormal items of OF and PF. Mild outliers are marked with a circle “o”. Extreme outliers are marked with an asterisk “*”.

Logistic regression analysis revealed that in a crude model, PF could be predicted by OF subdomains of difficulty eating hard food (OR = 3.37, 95% CI = 2.02–5.63; *p* = 0.000), choking (OR = 3.65, 95% CI = 1.91–6.97; *p* = 0.000), denture use (OR = 2.96, 95% CI = 1.78–5.17; *p* = 0.000), inability to chew hard food (OR = 3.8, 95% CI = 2.18–6.62; *p* = 0.000), and high risk of OF (OR = 4.56, 95% CI = 2.5–8.32; *p* = 0.000). In the adjusted model, PF was significantly associated with the OF subdomains of difficulty eating hard food (OR = 2.17, 95% CI = 1.21–3.88; *p* = 0.009), choking (OR = 2.64, 95% CI = 1.29–5.43; *p* = 0.008), denture use (OR = 2.44, 95% CI = 1.26–4.17; *p* = 0.008), inability to chew hard food (OR = 2.27, 95% CI = 1.48–5.0; *p* = 0.001), and high risk of OF (OR = 3.03, 95% CI = 1.51–6.1; *p* = 0.002) (Table 3). A high mean score and mean abnormal items with OF presented severe PF status, and a linear correlation of OF and PF was found. The higher levels of frailty were associated with higher mean score and mean abnormal items of OF (Figure 1 and Figure 2).

The adjusted model was adjusted for age, number of natural teeth, self-reported health status, Parkinson’s disease, and CDR score. CI, confidence interval; OR, odds ratio.

A regression model was constructed in eight steps and reduced from the original 13 variables to six (Table 4). The model with five indispensable variables had a predictive value of 78.2%. The five predictors for PF in this study were the following: age (*p*= 0.008), self-reported health status of fair (*p* = 0.000) and poor (*p* = 0.000), and mild cognitive impairment (*p* < 0.001) and denture use (*p* = 0.011).

## 4. Discussion

In this study, we investigated the association between OF and PF among rural middle-old community-dwelling people with cognitive decline. In addition, we examined the association of the characteristics of middle-old people, self-reported health status, OF subdomains, OF risk, and PF with PF predictors. In this study, 60.4% of the population had a high risk of OF, and 80.7% of elderly people had pre-frailty to frailty status. PF risk was associated with OF subdomains (difficulty eating hard food, choking, denture use, and inability to chew hard food) and high risk of OF.

Comparing the different models in this study, the OR for the inability to chew hard food was higher than for other OF subdomains, which is a crucial indicator for OF evaluation. Difficulty in eating was associated with slow gait speed, increased frailty risk, and mobility limitations [21]. Limited oral chewing function, which may worsen nutrient absorption status, may lead to PF [22]. The choking item in the OF subdomain was also a predictor of PF in this study. A study reported that frailty was associated with choking history (OR = 2.954), whereas drinking was associated with dysphagia [23]. Swallowing impairments affect the ability to drink liquids and result in aspiration risk in frail elderly people [24]. Poor swallowing function with age affects nutritional status and frailty; a decreased nutritional score and body weight, decreased chewing function, and decreased water drinking function were frailty predictors [25]. In this study, middle-old people who used dentures had a high risk of PF. Denture use was found to be a predictor for PF. A study in Mexico indicated that the use of complete dentures among elderly people increased the risks of malnutrition, weight loss, and components of frailty syndrome [26]. Another study showed that people aged ≥60 years with <20 teeth had a high risk of pre-frailty or frailty irrespective of whether they used dentures [27]. Therefore, whether using a partial or complete denture predicts PF in rural middle-old people with cognitive decline must be further explored.

In the present study, a linear correlation of OF mean score and PF was found. The adjusted model showed that elderly people with high risk of OF had an OR of 3.03 for PF. One study showed that OF was associated with poor gait performance among community-dwelling older adults [28]. The association between OF and nutritional status was reported in a Japanese study of older adults with mean age = 77 years; OF had higher odds of more severe malnutrition (adjusted OR = 2.17). [29]. A Japanese study presented that OF is related to frailty status, general health and nutrition [30]. Therefore, early detection of OF in rural middle-old community-dwelling people and using the OF assessment method for easy, rapid, and feasible improvements in OF and PF in community and clinical practice were important.

Differences in the demographic data between those with no frailty, pre-frailty and frailty status were compared in this study. The mean CDR scores for elderly people at different frailty levels exhibited significant differences. Mild cognitive decline was found to be an indispensable predictor for PF. Frailty was independently associated with global cognition and dementia status [31]. PF increased dementia risk for people with cognitive impairment and poor general health [32]. The relationship between frailty and incident dementia was significant for adults with global cognitive function in the upper three quartiles (hazard rate: 3.48 95%CI [1.98–6.12]) [33]. Communication difficulties are a consequence of nerve cell failure, and people with cognitive decline present difficulty in understanding, verbally expressing, repeating, reading, and writing [34]. Self-reported health status levels of fair and poor were also found to be indispensable predictors for PF in this study. Self-perceived oral health with restricted food types and dental status was significantly associated with PF [35]. Elderly people with cognitive decline may require guidance for self-reported oral frailty problems, which could improve their oral health. Therefore, further research should use physiological monitoring equipment to understand OF and PF in elderly people with moderate to severe cognitive decline.

Oral status may be an essential contributor to Alzheimer’s disease and late-life cognitive disorders, and oral microbiota and tooth loss are also associated with an increased risk of Alzheimer’s disease [36]. Oral health management could improve not only oral status, swallowing function, and nutritional status but also activities of daily living and in-hospital mortality [37]. Worsening frailty condition must be prevented in the elderly population with cognitive impairment. Therefore, novel strategies must be used, and oral function and nutritional status must be maintained or improved to reduce the burden of both oral dysfunction and frailty [38].

Age-related differences in oral function were found in older adults. Elderly people with frailty status had significantly poorer oral function than pre-frailty and robust elderly people [39]. In the present study, middle-old people with an average age of 81.8 years had a higher risk of OF, and the proportion of this population with frailty was high. Older people with Parkinson’s disease between different frailty levels presented significant differences in this study. Parkinson’s disease restricts physical motor function, which in turn leads to sarcopenia or PF [40,41,42]. PF risk (OR = 2.40) and gait speed (OR = 0.85) were associated with OF. PF is closely related to OF, and decreased gait speed is a crucial indicator of OF [43].

This study had four limitations. First, 57 potential participants were excluded because they did not utilize the community care center again and could not complete the assessment; Second, 75% of females in the study were involved in community-based cognitive decline care centers; the life expectancy for women in Taiwan exceeds the global average by 9.2 years, and researchers estimate that cognitive function in women declines faster with age [44]. Third, the sample consisted of middle-old people from rural communities; therefore, the research results cannot be generalized to other populations. Fourth, the nature of the cross-sectional study made causal inference impossible.

## 5. Conclusions

Although this study has some limitations, the findings demonstrated that age, self-reported health, mild cognitive impairment, and the OF subdomain of denture use could predict PF. OF can be attributed to various chronic diseases, oral health, and oral health problems. Early screening, assessment, and self-management of oral frailty in rural middle-old community-dwelling people with cognitive decline are essential to promote oral health, which can be conducted through programs such as an interdisciplinary oral care program for oral care in chronic diseases. Oral rehabilitation, oral health promotion education, and nurse–medical-dental collaboration may be effective interventions for preventing OF in elderly people with cognitive decline. Our findings suggest that early detection of OF, routine OF assessment and care for the elderly population with cognitive decline in rural community-based care centers, and prevention of OF at an early stage of cognitive decline are essential for healthy aging. OF is a crucial indicator of PF development, but a longitudinal study is needed to analyze the causal relationship between OF and PF.

## Figures and Tables

**Table 1:** Characteristics of study participants stratified by frailty level.

Variables	Overall (*n* = 308)	Robust (*n* = 93)	Pre-Frailty (*n* = 147)	Frailty (*n* = 68)	*p*-Value
Age, years, mean (SD)	79.7 (7.2)	77.6 (7.3)	80.1 (6.9)	81.8 (7)	0.001 ^b^
Gender, female, *n* (%)	231 (75)	67 (72)	112 (76.2)	52 (76.5)	0.732 ^a^
Number of natural teeth, mean (SD)	7.8 (9.3)	10.7 (10)	7.4 (9.2)	4.8 (7.5)	<0.001 ^b^
Education level, *n* (%)					
Elementary school and below	253 (82.1)	74 (79.6)	120 (81.6)	59 (86.8)	0.882 ^a^
Junior high school and above	54 (17.5)	19 (20.4)	26 (17.7)	9 (13.2)	
Number of chronic diseases, mean (SD)	1.5 (1.1)	1.4 (1.1)	1.5 (1.2)	1.7 (1.1)	0.133 ^b^
Hypertension (yes), *n* (%)	176 (57.1)	51 (54.8)	87 (59.2)	38 (55.9)	0.780 ^a^
Diabetes mellitus (yes), *n* (%)	64 (20.8)	20 (21.5)	33 (22.4)	11 (16.2)	0.562 ^a^
Heart disease (yes), *n* (%)	41 (13.3)	10 (10.8)	18 (12.2)	13 (19.1)	0.265 ^a^
Parkinson’s disease (yes), *n* (%)	16 (5.2)	2 (2.2)	6 (4.1)	8 (11.8)	0.018 ^a^
Self-reported health status (yes), *n* (%)					
Good, *n* (%)	50 (16.2)	28 (30.1)	20 (13.6)	2 (2.9)	<0.001 ^a^
Fair, *n* (%)	172 (55.8)	55 (59.1)	90 (61.2)	27 (39.7)	
Poor, *n* (%)	86 (27.9)	10 (10.8)	37 (25.2)	39 (57.4)	
CDR score, mean (SD)	0.87 (0.53)	0.75 (0.5)	0.92 (0.54)	0.93 (0.52)	0.035 ^b^
CDR, *n* (%)					
Questionable dementia	171 (55.5)	68 (73.1)	72 (49)	31 (45.6)	0.002 ^a^
Mild cognitive impairment	93 (30.2)	15 (16.1)	52 (35.4)	26 (38.2)	
At least moderate cognitive impairment	44 (14.3)	10 (10.8)	23 (15.6)	11 (16.2)	

SD, standard deviation; CDR, clinical dementia rating. Mean ± SD for continuous variables, and number and percentage for categorical variables. ^a^ χ^2^ test for proportions and nominal variables. ^b^ Based on one-way analysis of variance.

**Table 2:** OF score and subdomains for middle-old people with different physical frailty statuses.

Variables	Overall (*n* = 308)	Robust (*n* = 93)	Pre-Frailty (*n* = 147)	Frailty (*n* = 68)	*p*-Value
OF abnormal items, mean (SD)	3.3 (1.6)	2.5 (1.4)	3.4 (1.6)	4 (1.6)	0.000 ^b^
OF score, mean (SD)	4.87 (2.51)	3.61 (2.18)	5.1 (2.46)	6.1 (2.32)	0.000 ^b^
Difficulty eating hard food (yes), *n* (%)	167 (54.2)	32 (34.4)	83 (56.5)	52 (76.5)	0.000 ^a^
Choking (yes), *n* (%)	94 (30.5)	14 (15.1)	49 (33.3)	31 (45.6)	0.000 ^a^
Denture use (yes), *n* (%)	238 (77.3)	58 (62.4)	122 (83)	58 (85.3)	0.000 ^a^
Xerostomia (yes), *n* (%)	79 (25.6)	16 (17.2)	42 (28.6)	21 (30.9)	0.078 ^a^
Less frequently going out (yes), *n* (%)	16 (5.2)	4 (4.3)	9 (6.1)	3 (4.4)	0.782 ^a^
Inability to chew hard food (yes), *n* (%)	135 (43.8)	22 (23.7)	70 (47.6)	43 (63.2)	0.000 ^a^
Brushing teeth < 2 times a day (yes), *n* (%)	65 (21.1)	22 (23.7)	31 (21.1)	12 (17.6)	0.653 ^a^
Visiting the dentist irregularly (yes), *n* (%)	209 (67.9)	66 (71)	89 (60.5)	54 (79.4)	0.017 ^a^
Risk of OF, *n* (%)					0.000 ^a^
Low risk	67 (21.8)	35 (37.6)	28 (19)	4 (5.9)	
Moderate risk	55 (17.9)	22 (23.7)	21 (14.3)	12 (17.6)	
High risk	186 (60.4)	36 (38.7)	98 (66.7)	52 (76.5)	

OF, oral frailty. Mean ± SD for continuous variables, and number and percentage of categorical variables. ^a^ χ^2^ test for proportions and nominal variables. ^b^ Based on one-way analysis of variance.

**Table 3:** Relationship among OF subdomains, risk of OF, and physical frailty.

	Crude Model			Adjusted Model		
	OR	95%CI	*p*-Value	OR	95%CI	*p*-Value
**OF subdomains**						
Difficulty eating hard food	3.37	(2.02–5.63)	0.000	2.17	(1.21–3.88)	0.009
Choking	3.65	(1.91–6.97)	0.000	2.64	(1.29–5.43)	0.008
Denture use	2.96	(1.7–5.17)	0.000	2.44	(1.26–4.71)	0.008
Inability to chew hard food	3.80	(2.18–6.62)	0.000	2.72	(1.48–5.0)	0.001
Visiting the dentist irregularly	3.37	(2.02–5.63)	0.442	1.51	(0.81–2.79)	0.194
**Risk of OF**						
Low risk	(Reference)				(Reference)	
Moderate risk	1.64	(0.8, 3.38)	0.179	1.62	(0.71–3.73)	0.254
High risk	4.56	(2.5, 8.32)	0.000	3.03	(1.51–6.1)	0.002

**Table 4:** The association of independent variables and physical frailty.

		B	SE	*p*-Value	OR (95%CI)
**Step 1**	Age	0.057	0.023	0.015	1.06 (1.01–1.11)
	Number of natural teeth	−0.002	0.017	0.912	1.0 (0.97–1.03)
	Number of chronic diseases	0.135	0.140	0.335	1.14 (0.87–1.51)
	Parkinson’s disease	0.400	0.828	0.629	1.49 (0.29–7.55)
	Self-reported health status				
	Good				
	Fair	1.419	0.403	0.000	4.13 (1.88–9.11)
	Poor	2.587	0.525	0.000	13.29 (4.75–37.22)
	CDR				
	Questionable dementia				
	Mild cognitive impairment	1.758	0.872	0.044	5.8 (1.05–32.03)
	At least moderate cognitive impairment	2.380	2.545	0.350	10.81 (0.07–1584.86)
	OF subdomains				
	Difficulty eating hard food (yes)	−0.260	0.581	0.655	0.77 (0.25–2.41)
	Choking (yes)	0.688	0.425	0.106	1.99 (0.86–4.57)
	Denture use (yes)	0.575	0.466	0.218	1.78 (0.71–4.43)
	Xerostomia (yes)	0.298	0.423	0.481	1.35 (0.59–3.08)
	Inability to chew hard food (yes)	0.409	0.435	0.347	1.51 (0.64–3.53)
	Visiting the dentist irregularly (yes)	−0.597	0.388	0.124	0.55 (0.26–1.18)
	Risk of OF				
	Low risk				
	Moderate risk	0.306	0.554	0.581	1.36 (0.46–4.02)
	High risk	0.726	0.821	0.376	2.07 (0.41–10.33)
**Step 9**	Age	0.057	0.021	0.008	1.06 (1.02–1.1)
	Self-reported health status				
	Good				
	Fair	1.388	0.393	0.000	4.01 (1.86–8.65)
	Poor	2.632	0.507	0.000	13.9 (5.14–37.56)
	CDR				
	Questionable dementia				
	Mild cognitive impairment	1.3250	0.372	0.000	3.76 (1.82–7.8)
	At least moderate cognitive impairment	0.667	0.456	0.143	1.95 (0.8–4.76)
	OF subdomains				
	Choking (yes)	0.703	0.399	0.078	2.02 (0.92–4.42)
	Denture use (yes)	0.858	0.335	0.011	2.36 (1.22–4.55)
	Inability to chew hard food (yes)	0.634	0.345	0.066	1.89 (0.96–3.71)

CI, confidence interval; OR, odds ratio.

## Data Availability

The data supporting the findings of the article can be obtained from the corresponding author on reasonable request.
